# Definition and Diagnosis of the Trigeminocardiac Reflex: A Grounded Theory Approach for an Update

**DOI:** 10.3389/fneur.2017.00533

**Published:** 2017-10-09

**Authors:** Cyrill Meuwly, Tumul Chowdhury, Nora Sandu, Eugene Golanov, Paul Erne, Thomas Rosemann, Bernhard Schaller

**Affiliations:** ^1^University Hospital Basel, Basel, Switzerland; ^2^Department of Anaesthesiology and Perioperative Medicine, University of Manitoba, Winnipeg, MB, Canada; ^3^Department of Pathology, University of Buenos Aires, Buenos Aires, Argentina; ^4^Department of Neurosurgery, Houston Methodist Hospital, Houston, TX, United States; ^5^Department of Primary Care, University of Zurich, Zürich, Switzerland

**Keywords:** reflex, trigeminocardiac, trigeminocardiac reflex, reflex, oculocardiac

## Abstract

**Background:**

The trigeminocardiac reflex (TCR) is defined as sudden onset of parasympathetic dysrhythmias including hemodynamic irregularities, apnea, and gastric hypermotility during stimulation of sensory branches of the trigeminal nerve. Since the first description of the TCR in 1999, there is an ongoing discussion about a more emergent clinical definition. In this work, the author worked out an approach to such an improved definition.

**Methods:**

In this study, a grounded theory approach was used. Literature about TCR was systematically identified through PubMed (MEDLINE), EMBASE (Ovid SP), and ISI Web of Sciences databases from 1/2005 until 8/2015. TCR was defined as a drop of heart rate (HR) below 60 bpm or 20% to the baseline. A grounded theory approach was used to analyze and interpret the data through a synthesis by the researcher’s perspectives, values, and positions.

**Results:**

Out of the included studies, the authors formed available data to an update of the understanding of changes in hemodynamic parameters (HR and blood pressure) in a TCR. According to this update, an HR deceleration should be a constant observation to identify a TCR episode while a drop in blood pressure should probably not being fixed to a certain percentage of decrease.

**Conclusion:**

The here presented working definition improves our understanding of the TCR. It leads the way to a new understanding of the TCR for a proper clinical definition.

## Introduction

The trigeminocardiac reflex (TCR) is a phylogenetic old reflex that manifests as sudden onset of changes in hemodynamic parameters, such as heart rate (HR) and mean arterial blood pressure (MABP), but also apnea and gastric hypermotility during stimulation of any branches of the trigeminal nerve. First observed by Kratschmer in 1870 (1) the *peripheral* variant of the TCR was establishment as Aschner–Dagnini reflex (and later known as oculocardiac reflex) by Aschner and Dagnini in the year 1908 (2). The other pioneers of this field include Kumada and colleagues (described trigeminal-depression response) (3), Shelly and Church (coined the term TCR), both working on animal models. The senior author first described and established the *central* TCR manifestation in humans during surgery in the cerebellopontine angle in the year 1999 (4). These authors showed a relevant drop of more than 20% in MABP and HR after central stimulation of the trigeminal nerve in 14 out of 125 patients (11%) who underwent tumor surgery in the cerebellopontine angle. In this work, it is given a structured explanation and a relatively simple, but emergent definition of the TCR, therefore, working out—for the first time—the basis to compare further studies (4). Schaller and colleagues also examined the TCR in various clinical variants during the following years (5–23) and developed the nowadays-established classification between the peripheral, central, and ganglion subtype of TCR (24). In this structured classification, the generally best-known oculocardiac reflex is included as a peripheral subtype of the TCR. Since the publication of these first articles in 1999, the better and better-examined phenomenon gained increased interest in neurosurgery and neuroanesthesia, demonstrating in the still gaining number of publications. However, since the first day of research, there does still exist now different new variants of the clinical definition of the TCR as these were all developed on an emergent basis during the last nearly 20 years. Now as more and more papers are describing the various clinical subtypes as well as a underestimated chronic subform of TCR, it is imperative to look for a more detailed definition of TCR again on a more systematic approach and to close this gap in the literature. The aim of this work is to develop, by the help of a grounded theory approach, a working clinical definition that is applicable to all subtypes of TCR.

## Methodology

To gain a better definition of the TCR, a grounded theory approach was used. Wolfswinkel et al. reported about the practical appliance of the grounded theory approach advanced by Strauss and Corbin (25, 26) in the methodical reviewing literature (27) as it is found in the growing number of literature on TCR. They described, “applying grounded theory aims to point to well-rooted and fruitful new links between variables” (p. 2). To gain such more “fruitful new links between variables” we choose the grounded theory literature review method since our objective was to close the gap in the literature and find out a more balanced definition of the TCR that includes all newly found TCR subtypes.

We considered four key issues, namely, sampling, creativity, reflexivity, and precision, as pointed out by Cutcliffe (28) to be fundamental to the use of grounded theory as a research method. However, to create the grounded theory literature review method, the five stage process that Wolfswinkel et al. (27) proposed was adopted: “define,” “search,” “select” (as all three covered by the literature research) “analyze” and “present” (as both presented in the Section “Result”).

### Literature Research

The authors conducted a systematic literature research in PubMed (MEDLINE), EMBASE (Ovid SP), and ISI Web of Sciences databases from 1/2005 until 8/2015. Search terms included “Trigeminocardiac reflex,” “Trigemino-cardiac reflex,” “Trigeminal depressor response,” and “Oculocardiac reflex.” All publications were included, and reference lists of all included articles were reviewed to identify additional relevant articles.

#### Definition of the TCR

We defined the occurrence of a TCR episode for the non-restrictive purpose of this study as *bradycardia*; a drop of HR below of 60/min or 20% or more from the baseline and/or asystole. Further, a TCR had to occur in a clinical setting during the surgery and fulfill at least one of the major criteria for a TCR as earlier defined by the authors (plausibility or reversibility) (4, 24). There is a strong need for a detailed description and comprehensible cause–effect relationship (4, 24), for every included case. But for the retrospective design of this study and to consider the different clinical manifestation of the TCR subtypes, hypotension, thus a drop of RR below 90/60 mmHg, 70 mmHg MABP, respectively, was an optional criterion and not required for inclusion.

Additional sample inclusion criteria beyond the time limits and search terms already mentioned included type of article, age, and language: case report or a case series with patients’ age from 1 to 99 years old and that are published in English, German, or French. We included all articles that reported a TCR in a clinical setting during surgery as defined earlier. If there was no link to a full-text version available, we tried to contact the author directly; if not successful, we excluded the article. All TCR cases were checked for double publication.

The primary search revealed 486 studies. After having used the inclusion criteria on the primary search result, a total of 45 studies remained to be included in this study.

### Grounded Theory

The constructivist grounded theory approach was used (29). Data used through the evaluation process were mainly collected in the literature research. Further opinion leader’s ideas and researchers own thinking concepts, developed through working since years in this field, were gathered. Through the whole working process, we noted our ideas, impressions, and concepts in a study diary. The collected data underwent an open, axial, and selective coding process. Out of this process results a model that is developed through a seesaw change between concrete data and a model level, between inductive and deductive thought and between open, axial, and selective codes. Data are therefore co-constructed and colored by the researcher’s perspectives, values, and positions. This position takes a middle ground between the realist and postmodernist positions by assuming an “obdurate reality” at the same time as it assumes multiple realities and multiple perspectives on these realities (30).

We have therefore performed the literature review as described earlier. The literature review was completed with summarizing the inadequacies of theoretical frameworks to explain what is going on in the proposed area of study. Then, there was a need of seeking new bodies of literature previously not explored to close the gaps.

### Covered Topics

The principal goal of this research was to close the literature gap and to create a working definition that leads to an updated model of TCR definition. It includes all newly described subtypes and new knowledge to the physiological background of this model.

## Results

The emerging results based on this grounded theory approach are presented in the following separated by the different topics covered.

### The Reflex Arc of the TCR—A Unique Entity

The TCR is triggered by physical and/or chemical stimulation of any of the nerve endings of branches of the fifth cranial nerve. After activation of sensory fibers, the afferent signal is transmitted through the Gasserian ganglion to the sensory nucleus of the trigeminal nerve, located on the floor of the fourth ventricle. The link between the afferent and efferent pathway form small internuncial fibers of the reticular formation; they connect the sensory nucleus of cranial nerve V with the efferent premotor neurons, located primarily in the nucleus ambiguous and the dorsal motor nucleus of vagus nerve (31). Completing this pathway, the activation of cardioinhibitory parasympathetic vagal neurons (5, 32) leads to the clinical manifestation. The connection between afferent and efferent fibers is presumably fully located in the brainstem as experimental studies with decerebrated animals showed a likewise trigeminocardiac potential (5, 33). However, in regard to the afferent pathway, there exist marked differences in subtypes of TCR, which lead to different reflex arcs. The peripherally stimulated TCR is related primarily *via* the spinal nucleus of the trigeminal nerve to the Köliker–Fuse nucleus, whereas the centrally stimulated TCR is conveyed *via* the nucleus of the solitary tract to the lateral parabrachial nucleus (31, 34). Previous studies showed, for the peripheral subtype (see below), a co-activation of the parasympathetic and sympathetic nervous system (35). These findings lead to a model where co-activation results in (sympathetically mediated) normo-/hypertension and (parasympathetically mediated) bradycardia. In this model, in a central subtype is no or at least less strong activation of the sympathetically nerve system (23, 24). This phenomenon explains the classic clinical manifestation, described in 1999 by the senior author (4). It seems that a defect interaction of neurons might play a principal role to be prone to the TCR (36) as it was already suggested in case of the SIDS (37).

### Updated Categorization of TCR Leading to a New Working Definition

As an update to the classic categorization that divides the TCR into central, peripheral, and ganglion subtype (24), the updated categories of the TCR are still based on the trigger point’s location (23). But a trigger point central to the Gasserian ganglion is now subdivided into a central TCR and a brainstem TCR. De Jong et al. showed in animal studies that injection of noradrenaline bilateral of the brainstem, thus the area of nucleus tractus solitarii, results in a decrease of MABP and HR of anesthetized rats (38). It was also seen that the effect of injected noradrenaline was prevented by a previous injection of the α-adrenergic blocking agent phentolamine, at the same site. The authors, therefore, suggested an inhibitory role of α-adrenoceptors in the nucleus tractus solitarii in the central control of MABP. Further, in clinical settings, there are reports about patients with brainstem trauma suggesting that variances in MABP intervals require an intact brainstem, whereas low frequency ~0.06 ± 0.02 Hz BP rhythms may be preserved by sympathetic spinal circuitry (39–41). This hypothesis remains one of the black spots in TCR research, as, from a clinical point of view, such changes in the brainstem must be present in the TCR case, but it has never been proven.

Likewise, peripheral TCR has also shown a new category, furthermore peripheral, as the classic TCR. The latest research has shown similar reflex arches between TCR and diving reflex (DR) (37). The DR, another phylogenic old brainstem reflex, initiates breath holding, slowing down of HR, decreased cardiac output, peripheral vasoconstriction, and increased MABP (5, 42). Same as the TCR, the DR is often seen in newborns causing a decrease in HR (5–51%) by single facial submersion (43, 44). The DR is triggered by sensory input on the trigeminal nerve and therefore considered as a subgroup of the TCR. In contrast to the TCR, the DR manifests clinically, next to apnea and bradycardia, as hypertension due to a sympathetic vasoconstriction. These findings support the new working definition where a more peripheral trigger point of the trigeminal nerve around its course, results in a stronger activation of the sympathetic nervous system and therefore clinically presents as bradycardia with hypertension (24) or apnea (45).

#### The Spinal-Cardiac Reflex (SCR)—A New Phenomenon in the TCR Classification Scheme

Chowdhury and Schaller (46) recently described, for the first time, the existence of an SCR during direct or indirect manipulation of the spinal dura during spinal surgery. The mechanical stretch of the spinal dura with its intrinsic and extrinsic innervation was considered the most potent provoking factor in inciting this reflex. As in every reflex, also this SCR has a threshold from which it will be triggered, explaining that not every dural manipulation leads to a reflex response. However, such vasovagal reactions are also a substantial part of the manifestation of either pain/fear/other emotional factors and/or decrease in the venous return due to any reason. The usual manifestation of vasovagal includes bradycardia (parasympathetic activation) and/or hypotension (sympathetic inhibition). In clinical practice, SCR—another potential subgroup of the TCR—caused by acute distension of the dura mater (innervated by V1) other than surgical manipulation, e.g., after aneurysm rupture, can play an important role on the high mortality manifesting early after SAH (42, 47, 48). Based on several observations, it seems that sudden decrease of venous return can activate such pathway (49, 50).

#### The Definition Criteria for TCR

Trigeminocardiac reflex is commonly defined as a sudden drop in HR and MABP of more than 20% as compared with the baseline values (4). Some articles examined the TCR with less restrictive definitions and asked of an observed reflex accompanied by 10% change in HR and MABP to the baseline (51). As a result of the ongoing discussion about the clinical definition of the TCR, according to a cause–effect relationship, our author group showed in previous research (4, 52) four definition criteria that allow identifying a sudden change in the hemodynamic parameter as TCR (24). We, therefore, defined *two major* (plausibility and reversibility) and *two minor* (repetition and prevention) criteria. An estimated TCR event should fulfill these two principal criteria, but not all the criteria must/can always be required to confirm a TCR. However, the more criteria are present; the more confirmed is a TCR. On the one hand, plausibility requires the event as a direct result of a physical or chemical manipulation (stimulation) of the trigeminal nerve or its peripheral branches. There has to be a direct link in time and action between stimulation and reaction (hemodynamic changes, apnea, and gastric hypermotility). On the other hand, reversibility describes that cease of the inducing stimulus should result in termination of the TCR event. However, in a clinical setting, there are some reports about continuing asystole after ceasing of manipulation that required cardiopulmonary reanimation maybe in a matter of a “point of no return” phenomenon (53). According to the minor criteria, a repetition of the stimulus should provoke the reflex each time, as a matter of ethics, this criterion cannot be evaluated in every TCR case in daily practice but is observed under some circumstances (19, 54). As seen in practice, a lighter and tender manipulation of the fifth cranial nerve can either prevent the occurrence of a TCR or result in less severe symptoms, what is, therefore, the most important preventing procedure. Other, not absolute methods include blocking of the nerve by local anesthetics or prior administration of anticholinergic drugs. It is important to understand that the minor criteria are no absolute criteria because neither repetition nor prevention can be fully guaranteed neither from a clinical nor a pathophysiological point of view.

### Clinical Example

The following clinical case is already published (21) but underlines that our new working definition improves the description of the phenomenon TCR.

An otherwise healthy young patient suffered from a trauma on the left eye and developed upper gaze diplopia. A computed tomography scan revealed a fracture of the left-sided orbital floor and minimal soft tissue entrapment. At that time, the patient was managed conservatively. After 1 month, the patient complained of several episodes of dizziness while looking up, palpitations, and having left-sided chest pain. Holter monitoring showed normal sinus rhythm but had multiple premature ventricular complexes. Stress electrocardiography and echocardiography also revealed no abnormality. The patient also started to experience slowing of his pulse rate during sleep and rest. On examination, his resting HR and blood pressure (awake) were 60 bpm and 130/80 mmHg, respectively, and reduced to 50 bpm and 100/60 mmHg, respectively, during sleep. The patient also complained of several episodes of interrupted sleep due to the sudden fluctuation of HR and MABP. These symptoms became progressively worse and after a few months, the patient was operated on for the left-sided fractures of the orbital floor. A couple of days after surgery, the patient’s cardiovascular symptoms improved dramatically.

#### Interpretation

From the clinical course, the here described case of an orbital fracture represents a clear TCR. However, based on the strict definition criteria of 1999 (4) (20% decrease of HR and MABP; cause–effect relationship), a TCR cannot be diagnosed as nocturnal sleep is characterized by a (physiological) prolonged blood pressure decrease. With the more flexible working definition (two major and two minor criteria), a TCR can be diagnosed as three (plausibility, reversibility, and repetition) out of four criteria are fulfilled.

## Discussion

The grounded theory approach has led to several important new insights into the definition of the TCR.

### A New Working Definition Is Needed

Regarding new findings in categorization and definition criteria, but also with the more and more diverse TCR-related phenomena (46, 55–57), it was evident, that the hitherto established definition of a strict 20% drop in HR and MABP was not suitable for all TCR types anymore. Current research leads to a more flexible working definition that is better adaptable to the now newly existent different TCR subtypes (22, 23, 31, 58, 59) and has helped to close this important gap in the literature.

#### The Role of HR—Updated

As seen in previous studies, a change in HR at TCR manifestation is described as deceleration in all subgroup of TCR except some reports about a TCR from the Ganglion subtype. No matter in which anatomical location the trigeminal nerve is stimulated, the vagal signal will lead to a slowdown of HR rarely resulting in asystole (22, 60–62). Pathophysiologically, this manifestation is explainable by a strong activation of the parasympathetic nervous system. Cardioinhibitory fibers that form the efferent part of the reflex arc (see above) connect the motor nucleus of the vagus nerve to the myocardium (61). These fibers have their main influence on the atrioventricular nodes and not on the ventricles and therefore a chronotropic effect. An activation of the vagal nerve, the cardioinhibitory fibers respectively, conduct an overbalance of the parasympathetic—over the sympathetic nervous system. As a result, the autonomic nervous system loses its ability to react to changes in MABP in a proper way to ensure the ejection volume for a sufficient distribution of blood. At this point, the TCR can also become symptomatic in conscious patients. These symptoms are mostly seen in the chronic manifestation of the TCR (21, 24, 63). According to the actual doctrine, a change in HR, a deceleration respectively, should be a constant observation to identify a TCR episode.

#### The Role of MABP—Updated

In an episode of TCR, the MABP is mostly known affected with hypotension. It has been seen that the anatomical location of stimulation around the course of the fifth cranial nerve has major influence if the MABP is increasing or decreasing (24). It seems like the more peripheral a stimulation of the trigeminal nerve, the more increasing and in return, the more central, the more decreasing is the MABP in a TCR episode (24). Highest blood pressures were observed in the DR as a—probably—very peripheral variant of the TCR (23, 37). In the DR, which is triggered by immersion of the head into the cold water, an obligatory increase of MABP (combined with an oxygen economizing bradycardia) is observed. The intensity of hemodynamic changes is proportionally changing with an increase of stimuli (e.g., water temperature) (37). Transforming these observations of MABP to defined criteria, a drop in MABP as criteria for TCR should probably not being fixed to a certain percentage of decrease (on the contrary to the accepted definition criteria of 20% decrease in HR and MABP). Moreover, should the identification of a TCR result out of a flexible surrogate definition model that includes all subtypes of TCR.

With the previous definition, often-reported hypertension in peripheral TRC cases forced those clinical presentations not to be considered as TCR although they fulfilled the major definition criteria (plausibility and reversibility). Further research with an accurate evaluation of changes in parameters during TCR episodes is needed to evaluate the here presented hypotheses that a drop in MABP is only facultative and it is required to develop a new working definition that is applicable in clinical and research setting.

### The Limitation of a New Surrogate Model

A model that is based on hemodynamic changes as HR and MABP is limited in the diagnostic reliability. A new, more flexible model includes all TCR episodes that fulfill the conservative inclusion criteria of a 20% drop of HR and MABP. With the adaption to the subtypes with less hypotension (e.g., peripheral and DR), new episodes will and can be identified and declared as TCR. This is, mostly from a clinical point of view, necessary for atypical but not less severe hemodynamic changes. The detection of slight manifestation of TCR, which resembles physiological changes in MABP/HR caused by changes in patients’ position, pain reactions, or drugs side-effects, is still a delicate challenge. A model that includes more cases shows automatically more false-positive episodes that are wrongly declared as TCR, while a strict model, on the other hand, risks excluding slight manifestation of TCR and thus underestimate the real number of TCR. A required change of 20% in HR seems from a clinical and research point of view still reasonable. If the here presented hypotheses can be proven in a systematic research, hypotension will further be a criterion to help to identify a potential TCR case but will not be a fundamental phenomenon.

A working definition always covers only a part of the reality, and the here presented attempt is no exception. Shortly, this working definition also needs to be proven in a clinical setting and, if necessary, adjusted to further findings. The main questions that have to be answered, if the here presented working definition is conceptually valid, i.e., do they share the different TCR symptoms as seen in various clinical settings and are they sufficiently reliable to allow consistent translational research. As developed from statistical studies, our model is more useful for a case series as for a single case. A single case is always complicated to put into a working definition; even the current definition does not exclude it.

### Practical Application of the Model

In daily practice, a TCR episode can cause severe bradycardia up to asystole and hypotension. Several reports of repetitive TCR in patients lead to the assumption that a more powerful trigger on the trigeminal nerve causes more noticeable hemodynamic changes (37, 54, 64, 65). As an only tender contact with the trigeminal nerve is one of the recommendations to prevent a TCR (31, 52), a diagnostic tool for upcoming, slight TCR episodes is essential. An early detection of TCR cases can help the anesthesiologist to warn the surgeon of probably more severe changes in hemodynamic parameters. The here presented working definition represents the basis of a new thinking model that contributes detecting TCR cases in daily practice. It separates TCR cases from another bradycardic phenomenon with other origins, also needing an entirely different treatment. In such a context, the current model has not only a great importance to refine further clinical studies but also to improve the treatment of our patients directly.

### View to the Future

The definition of the TCR is in a continual process. Further research about the hemodynamic changes related to the subtypes is necessary to develop and prove a surrogate definition model for clinical and research setting. As a prominent part of the nowadays knowledge results from analyses of case reports (66), further reports and meta-analyses have major priority to have further insights into the behavior of the TCR. There is a need for continuous flexibility in definition models, in this case, according to the location of the trigger point and the resulting subgroup that helped to explain further details of the TCR.

### Conclusion

By a grounded theory approach, the here presented working definition improves our understanding of the TCR; either in research as also in clinics. However, like every assumption, it cannot explain the whole phenomenon. But, the here presented model is a significant step to more detailed and precise understanding of the TCR.

## Author Contributions

CM and BS created the conception of the work and performed the data collection and the draft. Together with TC, they analyzed and interpreted the data. All the authors helped with critical revision and final approval.

## Conflict of Interest Statement

The authors declare that the research was conducted in the absence of any commercial or financial relationships that could be construed as a potential conflict of interest.
